# Effects of customized insoles with medial wedges on lower extremity kinematics and ultrasonographic findings in plantar fasciitis persons

**DOI:** 10.1038/s41598-023-35862-6

**Published:** 2023-05-27

**Authors:** Suthasinee Thong-On, Pavinee Harutaichun

**Affiliations:** Faculty of Physical Therapy, Mahidol University, 999 Phuttamonthon 4 Road, Salaya, Nakhon Pathom Thailand

**Keywords:** Diseases, Health care, Health occupations, Medical research, Mathematics and computing

## Abstract

The customized insole is widely recommended as an effective intervention for pain reduction and foot function improvement in plantar fasciitis persons. However, it is unclear whether the additional correction of medial wedges could change the kinematics from the only insole. The objectives of this study were thus to compare customized insoles with and without medial wedges on lower extremity kinematics during gait and to determine the short-term effects of the customized insole with medial wedges on pain intensity, foot function, and ultrasonographic findings in plantar fasciitis persons. A within-subject, randomized, crossover design within motion analysis research laboratory was conducted among 35 persons with plantar fasciitis. Main outcome measures included joint motions of the lower extremity and multi-segment foot, pain intensity, foot function, and ultrasonographic findings. The customized insole with medial wedges produced less knee motion in the transverse plane and hallux motion in all planes during the propulsive phase than that without medial wedges (all p < 0.05). After the 3-month follow-up, the insoles with medial wedges decreased pain intensity and increased foot function. Abnormal ultrasonographic findings also decreased significantly after the 3-month treatment of insoles with medial wedges. Customized insoles with medial wedges seem superior to those without medial wedges on both multi-segment foot motion and knee motion during propulsion. Positive outcomes from this study supported the use of customized insoles with medial wedges as an effective conservative treatment in patients with plantar fasciitis.

**Trial registration**: TCTR20210928006 (28/09/2021).

Plantar fasciitis (PF) is one of the most common foot and ankle problems, causing pain around the inferomedial aspect of the heel^1^. Patients with PF are prone to thickened plantar fascia and hypo-echogenicity from soft tissue edema on ultrasonographic (US) evaluation^2^. This anatomical impairment would lead to the loss of fascial elasticity, resulting in the reduction of medial arch height and excessive midfoot pronation^3,4^. Such abnormal movements may lead to functional biomechanical deficits of the lower extremity muscles, ankle joint, knee joint, and hip joint that could induce abnormal gait adaptation^5^, which were consistent with the theoretical causation of PF and other lower extremity overuse injuries.

Since the presence of PF involves both anatomical impairments of the plantar fascia and deviations in lower extremity biomechanics, the customized insole is thus widely recommended as an effective intervention for pain reduction and foot function improvement^1,6^. However, it is unclear whether the customized insole could provide positive effects on anatomical and biomechanical outcomes in PF persons, as most studies used subjective assessment to investigate the effectiveness of the customized insole^7–12^. From a systematic review, a previous study found a significant reduction in plantar fascia thickness in the insole group compared with the control group^13^. Regardless, there was a lack of studies that specifically evaluated the kinematic changes during gait from the customized insole in people with PF.

To date, three studies have reported kinematic changes from the customized insole in persons with foot pain, including midfoot osteoarthritis^14^ and symptomatic pronated foot^15,16^. Based on the previous findings, the customized insole with medial wedges at either the rearfoot or forefoot seemed to provide significant changes from the kinematic outcomes. Anyways, the angles of rearfoot and forefoot should be additionally examined to prescribe the appropriate amount of correction needed^17–21^. A previous study compared the lower extremity and multi-segment foot kinematics during the stance phase of gait between two different foot assessment techniques, including the intrinsic and extrinsic foot assessment techniques^20,22^, for the prescriptions of medial wedges among individuals with PF^23^. The results confirmed that both designs of medial wedges produced significant biomechanical changes when compared with shod during walking. Therefore, either design of medial wedges could be used for individuals with PF^23^. As the intrinsic foot assessment is widely used by podiatrists to determine the rearfoot and forefoot angles before customizing insole^17,24,25^, the present study used this technique to produce appropriate angle of medial wedges at the rearfoot and forefoot, which were put under the heat-molding customized insole. This study aimed to compare customized insoles with medial wedges and those without medial wedges on lower extremity and multi-segment foot kinematics during the stance phase of gait, as well as to determine the short-term effects of customized insoles with medial wedges on pain intensity, foot function, and US findings in individuals with PF.

## Methods

### Study design

A within-subject, randomized, crossover design in a biomechanical laboratory was used in this study. Three conditions included the shod condition, customized insole without medial wedges condition, and customized insole with medial wedges condition. Participants were additionally asked to use the customized insole with medial wedges and were followed up for 3 months. The research protocols were approved by the center of Ethical Reinforcement for Human Research of Mahidol University (COA No. MU-CIRB 2020/285.2109). All experiments were performed in accordance with relevant guidelines and regulations.

### Study participants

At least 27 participants had a power of 80% and a significance level of 5% to detect biomechanical differences among the three conditions with a medium effect (Cohen’s d = 0.5). Inclusion criteria were: (1) age between 18 and 60 years old; (2) specific criteria of PF^1^; (3) symptom of heel pain at least 6 weeks, indicating the chronic condition^26^; (4) average pain intensity during last week at least 30 mm on a 100-mm visual analog scale (VAS). The exclusion criteria were as follows: (1) body mass index (BMI) greater than 30 kg/m^2^; (2) leg length difference greater than 1 cm; (3) positive sciatica test, indicating L5-S1 nerve root irritation; (4) history of lower-extremity fracture; and (5) diagnosis of at least one disease as follows: gout, diabetic neuropathy, rheumatoid arthritis, systemic lupus erythematosus (SLE), cancer, infectious disease and tumor. Informed consent was obtained from all participants prior to data collection.

### Customized insole

**Figure 1:**
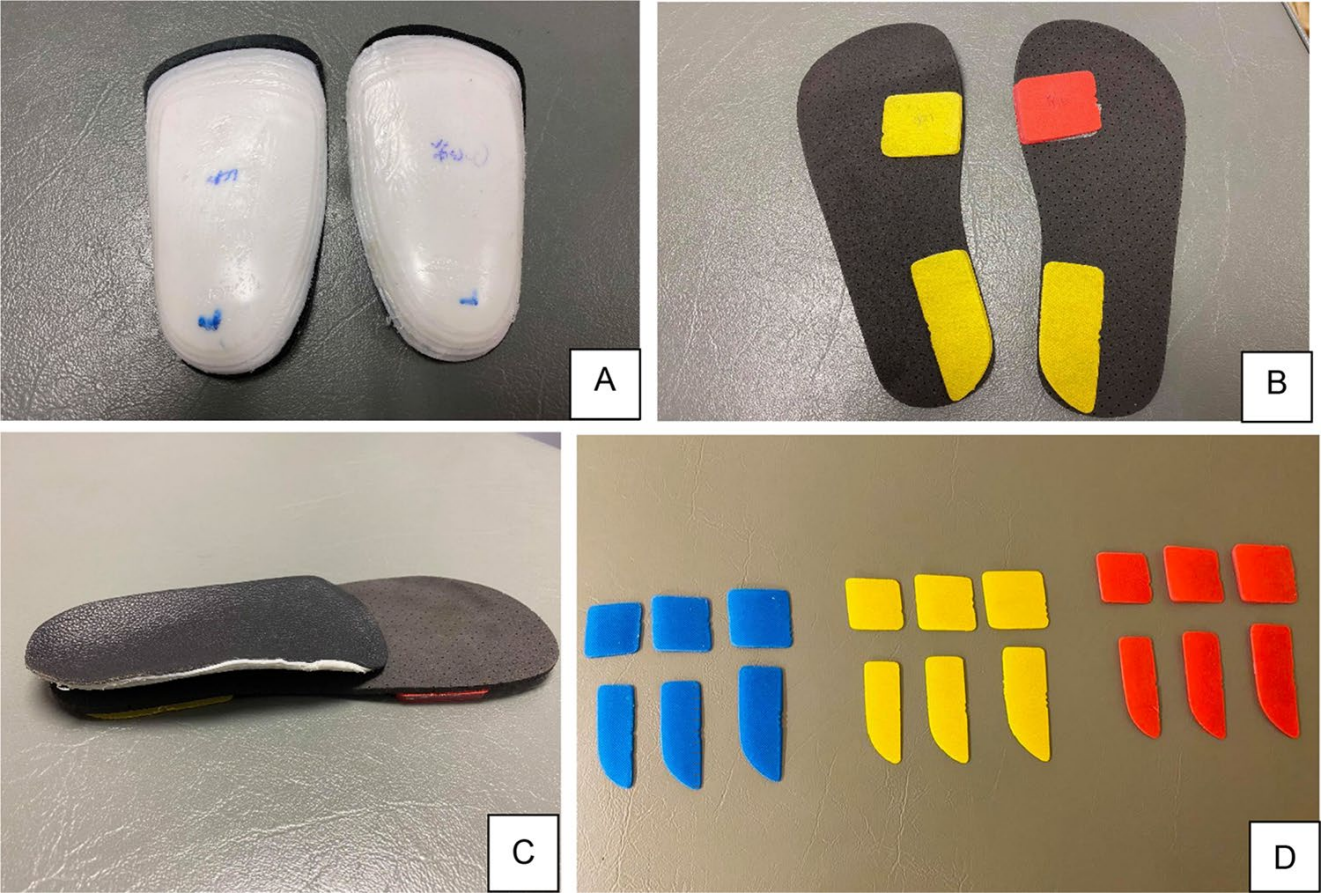
Customized insoles in the present study (**A** customized insole, **B** medial wedges with a full length of soft foam layer, **C** customized insole with medial wedges, **D** medial forefoot and rearfoot varus wedges with three different sizes i.e. small, medium, large. Blue color is the 3-degree wedge, Yellow color is the 6-degree wedge, Red color is the 8-degree wedge).

Participants were given one pair of customized insoles (Fig. 1A) from the 1st physical therapist who had 7 years of experience in the use of insoles for musculoskeletal problem management. It was a 3-quarter-length insole with three layers, which included one layer of 1.2-mm genuine leather to increase comfort and two layers of 1-mm polyvinyl chloride (PVC) to increase strength. It incorporated a heat-molding process, which could be set within approximately three minutes, to adjust the individual foot shape in the sitting position. There was no negative cast for the production of customized insole in this study. Anyways, the medial wedges were used to adjust the foot position of each participant following the foot assesment from Root^22^ in the subtalar neutral position. The forefoot angle in this position was used to determine the degrees of the rearfoot and forefoot varus wedges. Previous studies suggested 50% correction of the forefoot angle, up to a maximum of 6 degrees for the use of rearfoot varus wedge, and 60% correction of the forefoot angle, up to a maximum of 8 degrees for the use of forefoot varus wedge^19,27^. Medial wedges with full length of a 3-mm soft foam layer (Fig. 1B) were added under the customized insole (Fig. 1C). The present study developed 3-degree (blue color), 6-degree (yellow color), and 8-degree (red color) wedges for the rearfoot and forefoot (Fig. 1D). There were three sizes for these products, which included small, medium and large sizes, following the foot length of participants (Fig. 1D). Regarding the sizes of medial wedges, the Europe (EU) size of foot length was used to determine the appropriate size, which included the EU size 36–39 (small), size 40–42 (medium), and size 43–46 (large). The participants were asked to use the customized insole with medial wedge as often as possible during weight-bearing activities, and at least 4 h per day were required.

### Gait assessment

A three-dimensional motion analysis system (10 cameras, Vicon, Vantage V5 series, Oxford, UK) was used to track the lower extremities and multi-segment foot motions during gait at a sampling rate of 100 Hz. The cameras were synchronized with two force plates (AMTI, model OR6-7, USA), which were set to have a sampling rate of 1000 Hz on an 8-m walkway. All participants were instrumented with 42 retroreflective markers following the Plug-In-Gait (PIG) model and the Oxford Foot Model (OFM) by the 2nd physical therapist. Then, they were asked to walk with a pair of commercially athletic shoes (Adidas, Model: Duramo SL) and a shoe with two types of customized insoles, which included an insole with medial wedges and without medial wedges, in randomized orders. Before data collection in each condition, participants were asked to walk for approximately one minute to familiarize with each condition. Data were collected for 3–5 successful gait trials per condition with a self-selected speed. The comfort level was assessed after walking in each condition ranging from 0 to 10 points, and a higher score represented more comfort. Data from the motion analysis system were collected once at the beginning of the study.

### Clinical and US assessment

The visual analog scale (VAS), foot function index (FFI), and ultrasonographic assessment were collected at baseline and at the 3-month follow-up. A previous study reported minimally clinically important differences (MCIDs) of each standard questionnaire among patients with foot pain, with an MCID of 9 mm for VAS and an MCID of 6.5 points for FFI^28^.

An ultrasound machine (RUSI, model Affiniti 50, Philips, NV, USA) with a broadband linear array (Model L12-3) was additionally used by the 2nd physical therapist to assess edema of the plantar fascia on the symptomatic side. Participants were evaluated in the prone lying with a neutral position of the ankle joint. A probe that was adjusted at a depth of 3 cm was placed at the plantar side over the medial tubercle of the calcaneus^29,30^. Then, the examiner captured the plantar fascia at 5 mm from the anteroinferior aspect of the calcaneus and measured its thickness and echogenicity^26^. The apparent US and uncertain US groups were additionally classified in this study. The former group included persons who met one of the diagnostic criteria for PF, either plantar fascia thickness greater than 3.8 mm or hypo-echogenicity in the plantar fascia that represented those with plantar fascia edema, and the latter included persons without these abnormal findings^31^.

### Data processing

Joint kinematics were tracked using Nexus software (version 2.8.1) to determine the peak angles and motions of the pelvis, hip, knee, rearfoot (hindfoot relative to tibia), forefoot (forefoot relative to hindfoot), and hallux. The kinematic and kinetic data were filtered by the 4th-order zero-lag, low-pass Butterworth technique at cutoff frequencies of 6 Hz and 30 Hz, respectively. Initial contact and toe-off events for each foot were identified using vertical ground reaction force (GRF) data with a 10 N threshold. The stance phase of each foot was then normalized over a gait cycle by using custom MATLAB software (R2017a). Joint motion was defined as the difference between the maximum and minimum joint angles within each subphase of the stance, including the contact phase, midstance phase, and propulsive phase^32^. Two peaks of normalized GRF were determined in three directions, including anteroposterior, mediolateral, and vertical directions.

### Statistical analysis

Characteristic data from the symptomatic limb are shown as the mean ± standard deviation (SD). In case of bilateral symptomatic limbs, data from the more symptomatic was used. Three inferential statistics were analyzed in the present study. First, repeated-measures ANOVA was used to compare the peak angles and the joint motions of the lower extremity and multi-segment foot as well as two peaks of ground reaction force during gait among three conditions, including the shod condition, customized insole with medial wedges condition, and customized insole without medial wedges condition. Bonferroni post hoc test was used for pairwise comparison. Regarding the nonparametric data, the Friedman test and Wilcoxon signed-rank test were used to compare such outcomes. Second, either the paired sample t test or the Wilcoxon signed-rank test was used to determine pre-post changes in pain intensity, foot function, and plantar fascia thickness after using the insole for 3 months. Finally, the chi-square test was used to compare the number of persons with apparent US and hypo-echogenicity pre- and posttreatment. All statistical analyses were performed using SPSS software version 22.0 (IBM Statistics, USA), with a statistical significance level of *P* < 0.05. Effect sizes using Cohen’s d were calculated for all variables^33^.

### Ethical approval

The study protocol was approved by the center of Ethical Reinforcement for Human Research of Mahidol University (MU-CIRB 2020/285.2109). All participants were agreed to sign the consent form, and they were not involved in the design of the study or in the interpretation or translation of the study findings.

### Patient and public involvement

Participants were not involved in the design of the study or in the interpretation or translation of the study findings.

## Results

Thirty-five participants with PF (26 females and 9 males), with an average age and BMI of 40.14 years (SD 10.53) and 26.35 kg/m^2^ (SD 5.65), respectively, participated in this study. Of those, the median duration of symptoms was 5 months (IQR 20.50). The participants had average walking speed of 3.14 km/h (SD 0.36) and cadence of 96.27 steps per minute (SD 9.27). The symptomatic limb showed a higher forefoot varus angle than normative values, which has been reported in previous studies^19,27,34^. Higher degrees of rearfoot and forefoot wedges were placed under the involved side than under the uninvolved side. During the experiment, participants felt more comfortable after wearing the customized insole with or without medial wedges than shod (p < 0.001) (Table 1). The participants used the customized insole with average hours per day of 2.52 h (SD 2.17) throughout the three months of follow-up period.

**Table 1:** Participant characteristics (n = 35). Data are shown as mean (SD), median (IQR) or number (%).

Characteristics	Mean (SD)/number (%)
Age, years	40.14 (10.53)
BMI, kg/m^2^	26.35 (5.65)
Females, number (%)	27 (64.3)
Duration of PF symptoms, months	5 (IQR 20.50)
Walking speed, km/h	3.14 (0.36)
Cadence, steps/minute	96.27 (9.27)
Femoral anteversion angle, degrees	14.63 (2.79)
Tibial torsion angle, degrees	22.48 (4.09)
Ankle inversion angle, degrees	14.60 (5.91)
Ankle eversion angle, degrees	6.26 (2.70)
Rearfoot angle, degrees	5.14 (3.00)
Forefoot angle, degrees	17.69 (7.57)
Foot posture index (FPI), scores	3.81 (4.79)
Rearfoot varus wedge	
Involved side, degrees	4.64 (1.78)
Uninvolved side, degrees	3.89 (1.64)
Forefoot varus wedge	
Involved side, degrees	5.31 (1.81)
Uninvolved side, degrees	4.86 (2.32)
Comfort with foot orthoses (FOs)	
Shoe, points	5.62 (2.01)
Customized insole, points	7.25 (1.60)
Customized insole with medial wedge, points	7.28 (1.63)

*BMI* Body mass index.

**Figure 2:**
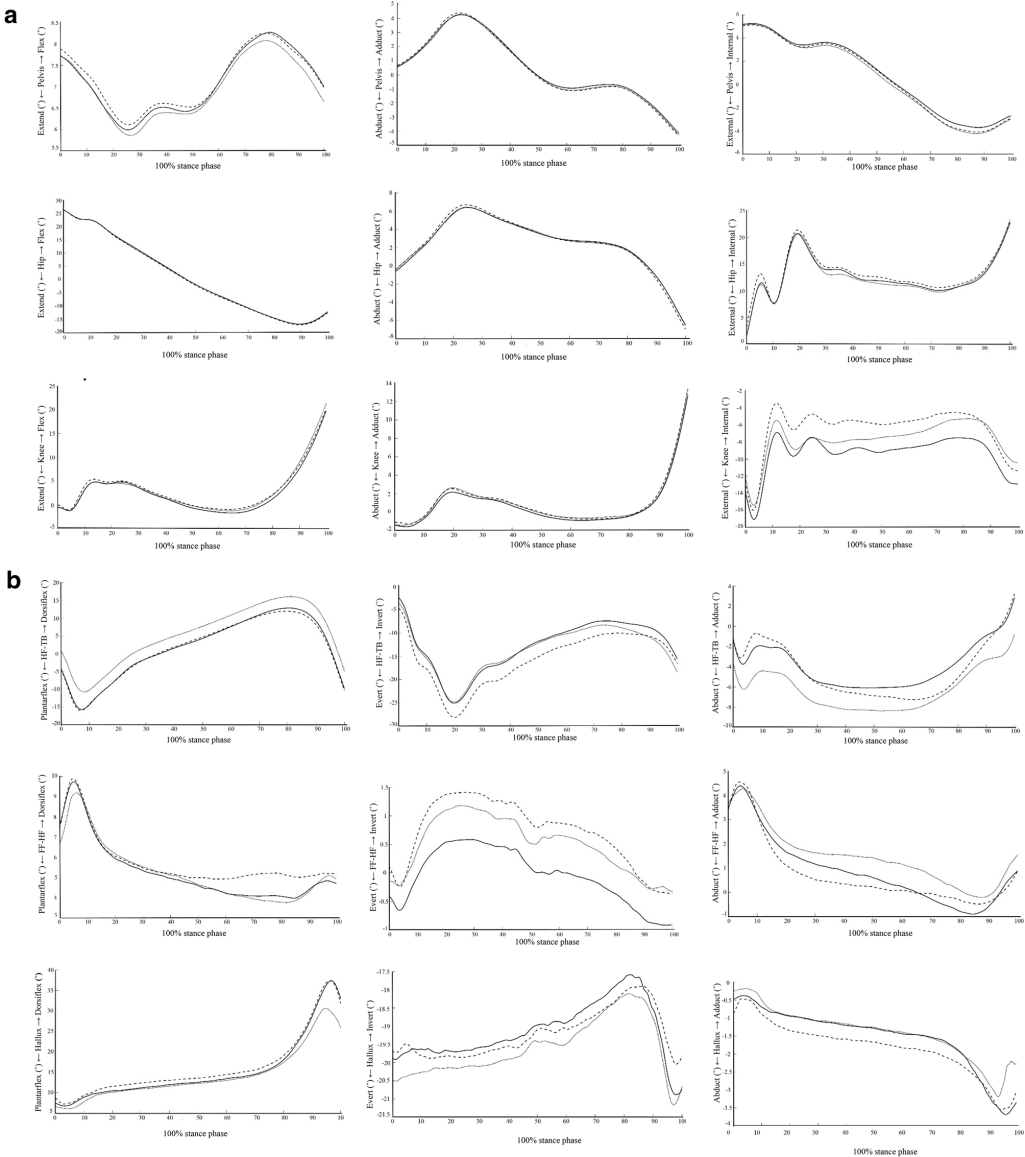
Comparisons of the lower-extremity kinematics among the shod condition, the customized insole without medial wedges condition, and the customized insole with medial wedges condition in each subphase of stance gait (n = 35) (Shod represented by a dashed line, Customized insole without medial wedges represented by a straighted line, and Customized insole with medial wedges represented by a dotted line).

Significant differences among the three conditions were found, with medium to large effects on the hip, knee, rearfoot, forefoot, and hallux motions during each subphase of the stance (Table 2, Fig. 2). When compared with shod and customized insole without medial wedge, the customized insole with medial wedge provided the least knee, forefoot, and hallux motions in the transverse plane (p = 0.004, p < 0.001, and p = 0.019, respectively), with large effect sizes seen during the contact phase, least rearfoot motion in the frontal plane (p = 0.028), with medium effect size seen during the midstance phase, least hip motion in the frontal plane (p = 0.035), with medium effect size, least knee motion in the transverse plane, least forefoot motion in the frontal plane, least hallux motions in the sagittal and frontal planes (p < 0.001, p < 0.001, p = 0.001, and p = 0.002, respectively), with large effect sizes seen during the propulsive phase.

**Table 2:** Comparisons of the mean (SD) or median (IQR) of the lower-extremity range of motion among the shod condition, the customized insole without medial wedges, and the customized insole with medial wedges in each subphase of stance gait (n = 35).

	Contact phase					Midstance phase					Propulsive phase				
	Mean (SD)/Median (IQR)			*P*	Effect size	Mean (SD)/Median (IQR)			*P*	Effect size	Mean (SD)/Median (IQR)			*P*	Effect size
	Shod	Insole no wedges	Insole with wedges			Shod	Insole no wedges	Insole with wedges			Shod	Insole no wedges	Insole with wedges		
Pelvis															
Sagittal (°)	1.9 (0.8)	2.0 (0.8)	1.9 (0.8)	0.955	0.063	2.8 (1.0)	2.8 (1.2)	2.7 (1.2)	0.576	0.238	2.0 (0.9)	2.0 (0.9)	1.9 (1.1)	0.729	0.180
Frontal (°)	4.0 (1.1)	3.8 (1.0)	3.9 (1.1)	0.525	0.271	6.0 (2.0)	5.7 (1.8)	5.7 (2.0)	0.078	0.519	3.9 (1.0)	4.0 (0.9)	3.9 (1.1)	0.934	0.090
Transverse (°)	2.2 (1.1)	2.2 (1.2)	2.3 (1.3)	0.950	0.063	7.8 (2.1)	7.6 (2.3)	7.3 (1.9)	0.247	0.403	2.1 [1.3,3.5]	2.0 [1.2,2.9]	2.1 [1.2,3.2]	0.416	0.423
Hip															
Sagittal (°)	12.3 (2.6)	11.8 (2.7)	12.4 (2.9)	0.095	0.519	28.9 (4.6)	28.7 (4.6)	28.4 (4.8)	0.452	0.293	8.7 (2.7)	8.7 (2.0)	8.7 (2.6)	0.989	0.063
Frontal (°)	7.1 (2.2)	6.7 (2.0)	6.8 (2.0)	0.240	0.403	5.4 (1.9)	4.9 (1.7)	5.1 (2.2)	0.120	0.478	10.5 (2.4)	10.3 (2.6)	9.9 (2.8)^‡^	0.035**	0.606
Transverse (°)	26.6 (9.3)	26.2 (9.8)	27.5 (10.1)	0.312	0.358	13.7 [10.1,17.0]	11.8 [10.2,14.1]	12.5 [10.2,16.3]	0.124	0.736	18.0 (9.6)	18.0 (9.8)	18.3 (10.7)	0.676	0.201
Knee															
Sagittal (°)	9.0 (3.7)	8.6 (3.4)	8.4 (3.6)	0.229	0.403	7.8 (2.6)	7.2 (2.5)	7.3 (2.4)	0.077	0.570	25.00 (8.9)	25.2 (9.2)	25.2 (9.4)	0.956	0.063
Frontal (°)	5.5 (2.1)	5.3 (2.0)	5.7 (2.5)	0.353	0.346	3.8 (1.8)	3.4 (1.2)	4.0 (2.0)	0.040**	0.674	22.3 (10.0)	21.7 (10.1)	22.0 (10.0)	0.557	0.247
Transverse (°)	15.9 (4.8)	14.9 (4.3)	13.9 (3.7)^‡^	0.004**	0.834	8.0 (2.7)	7.9 (3.0)	7.8 (2.8)	0.932	0.090	9.3 (4.1)	8.6 (4.1)	7.3 (3.8)^‡^,*	< 0.001**	1.044
Rearfoot															
Sagittal (°)	16.6 [13.4,17.9]	16.2 [14.0,18.7]	17.5 [15.1,16.6]	0.368	0.478	16.9 (5.1)	18.0 (4.3)	18.2 (4.3)	0.070	0.540	31.5 (6.7)	33.1 (7.1)	31.1 (7.8)	0.038**	0.617
Frontal (°)	25.5 [19.5,32.5]	25.5 [18.9,30.1]	23.2 [19.5,30.4]	0.828	0.203	18.3 (6.8)	18.0 (6.6)	16.8 (6.3)^‡^	0.028**	0.637	13.0 (7.0)	14.1 (7.1)	14.9 (7.0)^‡^	0.003**	0.810
Transverse (°)	9.9 (3.9)	11.1 (4.5)^†^	10.2 (3.9)	0.028**	0.656	7.3 (3.2)	7.7 (3.5)	7.4 (3.2)	0.686	0.211	15.5 (9.2)	15.9 (9.2)	14.7 (9.2)	0.301	0.358
Forefoot															
Sagittal (°)	4.8 (1.2)	5.0 (1.5)	4.8 (1.4)	0.627	0.220	2.1 [1.5,2.5]	1.9 [1.6,2.4]	2.3 [1.8,2.7]	0.572	0.369	2.7 (1.0)	2.7 (0.7)	2.5 (0.8)	0.607	0.247
Frontal (°)	1.9 [1.6,2.2]	1.5 [1.1,1.9]^†^	1.7 [1.4,2.0]^‡^	< 0.001**	1.970	1.4 [1.1,1.8]	1.4 [1.2,2.0]	1.5 [1.1,1.9]	0.433	0.436	1.7 (0.8)	1.4 (0.6)^†^	1.3 (0.7)^‡^	< 0.001**	1.149
Transverse (°)	4.0 (1.2)	3.1 (1.1)^†^	2.8 (0.9)^‡^	< 0.001**	1.865	2.0 (0.8)	2.2 (0.7)	2.0 (0.7)	0.173	0.454	3.6 (1.6)	3.5 (1.2)	3.5 (1.4)	0.896	0.110
Hallux															
Sagittal (°)	5.2 (2.9)	4.8 (2.8)	4.7 (2.5)	0.271	0.375	6.2 (3.1)	6.1 (3.1)	6.0 (3.2)	0.860	0.127	24.8 (8.2)	23.6 (7.0)	20.4 (7.4)^‡^,*	0.001**	0.950
Frontal	1.8 [1.3,2.4]	1.3 [1.1,2.1]^†^	1.3 [1.0,1.9]^‡^	0.038**	0.911	2.5 (1.4)	2.4 (1.1)	2.3 (0.9)	0.618	0.238	4.9 (1.8)	5.6 (1.6)	4.7 (1.5)*	0.002**	0.892
Transverse	0.8 [0.4,1.4]	0.7 [0.2,1.2]^†^	0.6 [0.2,1.0]^‡^	0.019**	1.029	0.7 (0.4)	0.7 (0.4)	0.7 (0.4)	0.857	0.142	2.5 (1.3)	2.7 (1.5)	2.3 (1.3)*	0.025**	0.674

^†^Significant difference between shod and insole without wedge; ^‡^Significant difference between shod and insole with wedge; *Significant difference between insole with and without wedge; **Significant difference of the main effect.

In addition, significant differences between the customized insole with and without medial wedge were found during the contact and propulsive phases. The insole with medial wedges provided superior effects than those without medial wedges on reduction of the peak ankle plantarflexion (p < 0.001) during the contact phase, knee motion in the transverse plane (p = 0.006), hallux motion in the sagittal, frontal and transverse planes (p = 0.008, p < 0.001 and p = 0.015, respectively), peak hallux dorsiflexion (p < 0.001) and peak hallux abduction (p = 0.002) during the propulsive phase. There were no effects of the customized insole on the two peaks of the ground reaction force in any direction (p ≥ 0.05). Data are shown in Tables 2 and 3.

**Table 3:** Comparisons of the mean (SD) or median (IQR) of the lower-extremity peak angle and normalized GRF among the shod condition, the customized insole without medial wedges, and the customized insole with medial wedges in the stance gait (n = 35).

	Condition			*P*	Effect size	P*	P**	P***
	Shod	Insole without wedges	Insole with wedges					
Hip								
Peak adduction (°)	6.95 (3.02)	6.49 (2.97)	6.58 (3.39)	0.154	0.449	N/A	N/A	N/A
Peak internal rotation (°)	25.00 [11.02, 35.77]	22.58 [8.72, 38.61]	27.65 [6.90, 39.43]	0.442	0.424	N/A	N/A	N/A
Knee								
Peak adduction (°)	3.41 [− 0.92, 7.16]	2.64 [− 2.47, 6.37]	3.07 [− 1.17, 6.84]	0.387	0.452	N/A	N/A	N/A
Peak internal rotation (°)	0.60 [− 13.21, 13.11]	− 0.15 [− 19.89, 7.74]	− 0.61 [− 21.59, 9.96]	0.067	0.816	N/A	N/A	N/A
Rearfoot								
Peak plantarflexion (°)	− 15.32 [− 21.33, − 10.18]	− 17.12 [− 22.20, − 12.32]	− 15.93 [− 19.96, − 9.09]	< 0.001	1.892	0.804	0.001	< 0.001
Peak eversion (°)	− 28.85 [− 48.77, − 4.96]	− 27.80 [− 49.00, − 4.02]	− 24.11 [− 44.12, − 7.17]	0.041	0.897	0.204	0.015	0.215
Peak abduction (°)	− 5.01 [− 8.59, − 1.96]	− 5.56 [− 8.53, − 3.35]	− 5.58 [− 8.56, − 3.06]	0.804	0.236	N/A	N/A	N/A
Forefoot								
Peak dorsiflexion (°)	7.18 [4.99, 17.40]	8.16 [5.33, 16.81]	7.69 [4.47, 15.86]	0.062	0.803	N/A	N/A	N/A
Peak inversion (°)	0.45 [− 1.46, 3.05]	0.43 [− 1.64, 2.37]	0.81 [− 1.21, 3.27]	0.014	1.080	0.021	0.491	0.089
Peak adduction (°)	3.5 [− 0.04, 7.48]	3.02 [− 0.47, 6.61]	3.86 [0.37, 6.08]	0.042	0.908	0.004	0.007	0.421
Hallux								
Peak dorsiflexion (°)	37.87 (13.53)	38.02 (13.12)	33.13 (12.22)	< 0.001	1.003	1.000	0.008	< 0.001
Peak inversion (°)	− 15.75 (9.32)	− 15.69 (9.30)	− 16.00 (8.94)	0.805	0.155	N/A	N/A	N/A
Peak abduction (°)	− 3.71 [− 4.78, − 0.50]	− 3.73 [− 5.72, − 0.70]	− 3.50 [− 5.19, − 0.87]	< 0.001	2.173	0.078	0.191	0.002
Anteroposterior GRF								
First peak	16.13 (3.99)	15.51 (3.88)	15.59 (3.78)	0.324	0.352	N/A	N/A	N/A
Second peak	21.15 (3.21)	20.62 (3.32)	20.55 (3.41)	0.159	0.444	N/A	N/A	N/A
Mediolateral GRF								
First peak	5.60 (1.38)	5.71 (1.45)	5.54 (1.53)	0.729	0.191	N/A	N/A	N/A
Second peak	4.38 (1.92)	4.47 (2.07)	4.03 (1.90)	0.057	0.565	N/A	N/A	N/A
Vertical GRF								
First peak	108.87 (8.85)	108.46 (9.53)	108.41 (9.30)	0.885	0.110	N/A	N/A	N/A
Second peak	105.24 (7.89)	105.24 (8.03)	104.65 (7.89)	0.808	0.155	N/A	N/A	N/A

*GRF* ground reaction force.

*Comparison between shod and insole without wedges; **Comparison between shod and insole with wedges; ***Comparison between Insole with and without wedges.

When compared with the shod, the customized insole with medial wedges decreased knee motion in the transverse plane (p = 0.006), forefoot motion in the frontal and transverse planes (p = 0.047 and p < 0.001, respectively), hallux motion in the frontal and transverse planes (p = 0.005 and p = 0.007, respectively), peak rearfoot eversion (p = 0.015), and peak forefoot adduction (p = 0.007) during the contact phase. It decreased rearfoot motion in the frontal plane (p = 0.041) during the midstance phase. In addition, the customized insole with medial wedges decreased hip motion in the frontal plane (p = 0.021), knee motion in the transverse plane (p = 0.001), hallux motion in the sagittal plane (p = 0.001), and peak hallux dorsiflexion (p = 0.008) during the propulsive phase.

Four participants dropped out from the follow-up; one felt uncomfortable from the insole, and three were under home quarantine due to COVID-19. After the 3-month follow-up, there were significant improvements, with large effects on morning pain, worst pain, and average pain during the last week (p < 0.001). Foot function scores were higher in all subscales, i.e., pain, disability, and activity limitation than baseline (p < 0.001). Regarding the US findings, there were significant reductions with large effect on the number of participants with hypo-echogenicity of the plantar fascia (p = 0.027) and that in the apparent US group (p = 0.023). However, data from plantar fascia thickness showed no significant difference. Data are shown in Table 4.

**Table 4:** Comparison of the median (IQR) or number (%) of the pain intensity, foot function index and US findings between pre- and post- 3 months after receiving the customized insole with medial wedges.

	Baseline (n = 35)	3-month follow up (n = 31)	*P*	Effect size
Pain intensity				
Morning pain, points	7.00 [4.00, 8.24]	2.50 [0.25, 6.00]	< 0.001*	2.880
Worst pain, points	7.00 [6.00, 8.24]	3.00 [1.00, 7.50]	< 0.001*	2.584
Average pain, points	5.00 [3.46, 7.00]	2.00 [1.00, 5.50]	0.008*	1.295
Foot function index				
Pain subscale, points	60.00 [38.57, 67.14]	15.56 [10.00, 47.78]	< 0.001*	3.420
Disability subscale, points	51.71 [33.21, 64.44]	13.33 [3.33, 50.00]	< 0.001*	3.101
Activity limitation subscale, points	24.00 [4.00, 35.00]	3.00 [0.00, 5.50]	0.005*	1.386
Total, %	47.14 [31.51, 58.00]	12.17 [7.28, 40.87]	< 0.001*	3.334
US Findings				
Apparent US, n (%)	24 (68.6%)	8 (25.8%)	0.023*	0.891
Hypoechogenicity, n (%)	20 (57.1%)	5 (16.1%)	0.027*	0.868
Plantar fascia thickness, mm	0.29 [0.24, 0.40]	0.31 [0.28, 0.37]	0.156	0.527

*US* ultrasonographic.

*Significant difference between pre- and post- 3 months.

## Discussion

The present findings supported most of the hypotheses that the customized insoles with medial wedge could change the kinematic variables of the rearfoot, forefoot, hallux, knee, and hip during the stance phase of walking. When compared with the customized insole without medial wedge, that with medial wedges demonstrated less knee motion in the transverse plane, less hallux motion in the sagittal, frontal and transverse planes than the other. A possible explanation for these findings may be related to the length of the customized insole. The present study used a three-quarter length customized insole, which uncovered the metatarsal heads of the forefoot. The addition of medial wedges, especially at the forefoot, to the customized insole could decrease overpronation at the subtalar joint^18,35^. Previous studies implied that persons with PF induced excessive foot pronation and over flattening of the medial longitudinal arch, resulting in greater loads on the plantar fascia than those without pain^36,37^. Such changes at the subtalar joint provided successive changes to the knee and hallux motions during propulsion^5^. As found in the present findings, the customized insole with medial wedges reduced the knee motion in the transverse plane and hallux motions in all planes when compared with that without medial wedges. The reduction in hallux motions has been previously indicated to reduce plantar fascia tension during late stance^38,39^. It thus seems that the customized insole with medial wedge provided superior effects than that without medial wedge on both multi-segment foot motion and knee motion during propulsion.

In addition, the present study demonstrated that the customized insole with medial wedge provided the least forefoot and hallux motions in the transverse plane during the contact phase, and the least rearfoot motion in the frontal plane during the midstance phase among the others. Since over movement of the foot in the frontal and transverse planes during the early phase of the stance is related to overpronation of the foot^40^, it was thus assumed from the findings that the insole with medial wedges could reduce excessive tension of the plantar fascia by decreasing over foot pronation from the early to mid-phase of the stance during gait. Other than the changes in multi-segment foot motions, the insole with medial wedges reduced the internal rotation of the knee during the contact phase when compared with the others. Biomechanically, the closed chain pronation of the foot produced a coupling mechanism, which induced internal rotation of the tibia via articulations at the subtalar and midtarsal joints^5^. Therefore, the functional changes from the uses of medial wedges could distribute the biomechanical alterations to the proximal structures. This in turn was temporally linked to hip and knee motions from the mid- to late-stance phase^5,39^. Although there was no significant difference in hip motion during the early phase, the insole with medial wedges condition demonstrated the least hip motion in the frontal plane and least knee motion in the transverse plane during the propulsive phase than the others.

A number of previous studies determined the kinematic changes of the medial wedge from the shod among persons with lower-extremity injuries, including patellofemoral osteoarthritis^41^, patellofemoral pain syndrome^42–44^, anterior knee pain^45^, and symptomatic pronated foot^15,16,46^. However, there was a lack of studies among persons with PF. Most studies used external posting ranging from 4 to 6 degrees of the medial wedge along with the full-length prefabricated insole^16,41,44–46^. The results from these studies showed no significant difference in hip and knee kinematics between the medial wedge and shod conditions. However, a study among persons with anterior knee pain found a significant reduction in only the peak ankle eversion in the medial-wedged condition when compared with the shod condition^45^. Similar to a study among persons with symptomatic pronated feet^15,16^, the researchers compared the peak rearfoot eversion between the customized insole with internal posting of the medial wedge and the shod conditions, and such outcome showed a significant reduction. Obviously, these studies did not separate the stance phase into subphases, and most studies provided the same degrees of the medial wedge to all participants^41–46^, which was not specific to the rearfoot and forefoot angles of each participant. Therefore, the present study showed different findings from those except for the significant finding of peak rearfoot eversion.

After the 3-month follow-up, the participants had significantly less pain intensity and higher foot function scores than at baseline. There was also significant reduction in the number of participants in the apparent group from the US findings, which included those with either plantar fascia thickness greater than 3.8 mm or hypo-echogenicity of the plantar fascia. Abnormal findings from ultrasonography represented perifascial fluid accumulation that caused plantar fascia edema in the symptomatic feet^47^. The risk of edema may be caused by stress repetition on the plantar fascia from overload weight bearing, such as running, obesity, foot deformities, and improper footwear^48,49^. The customized insole with a medial wedge could act as the passive structure of the plantar fascia that leads to a reduction in abnormal findings of the plantar fascia.

However, there was no significant difference regarding the plantar fascia thickness of the plantar fascia. Among 24 participants in the apparent US group, there were only 4 participants with plantar fascia thickness greater than 3.8 mm at baseline. After 3-month follow up, there were only 3 participants with that criteria from a total of 8 participants in the apparent US group. Obviously, an average thickness of the plantar fascia among participants is less than the diagnostic criteria for PF^31^. A previous study implied a significant effect between the gender and the plantar fascia thickness. The result demonstrated that the male persons with PF had significantly more plantar fascia thickness in the symptomatic side than the female^50^. There was the imbalance in the gender ratio in this study, with a higher number of the female participants. This might indicated the lower mean of the plantar fascia thickness in the present finding.

Some limitations should be concerned. For example, most participants in this study were obese, which could produce measurement error from skin movement. A pre-post experimental design was used to determine the clinical outcomes and US findings from the use of the customized insole with medial wedges. The control group should be included in further studies to compare such effects. And the therapist used a heat moulding method instead of a negative casting method. The present results could thus reflect the biomechanical effects of customized insole with only heat-moulding method and not other methods of production. However, several strengths must be acknowledged. The results could be generalized to PF population. Furthermore, this is the first study to determine the effects of customized insoles using joint kinematics and US diagnosis as objective outcomes among persons with PF. Although joint kinematics were only investigated as immediate effects, clinical outcome and US diagnosis were additionally determined as short-term effects. Positive outcomes from the present study supported the use of customized insoles with medial wedges as an effective conservative treatment in patients with PF. Further studies should use randomized controlled trials and extend the follow-up period. Joint kinetics and muscle activity should be additionally included to comprehensively investigate the biomechanical effects of the customized insole in individuals with PF.

## Conclusion

Customized insoles with medial wedges seem superior to those without medial wedges on both multi-segment foot motion and knee motion during the stance phase of gait. The former produced less knee motion in the transverse plane and hallux motion in all planes during the propulsive phase. When compared with the shod, the customized insole with medial wedges decreased knee motion in the transverse plane, rearfoot motion in the frontal plane, forefoot motion in the frontal and transverse planes and hallux motion in the frontal and transverse planes during the early to mid-phase of the stance. In addition, it decreased hip motion in the frontal plane, knee motion in the transverse plane, and hallux motion in the sagittal plane during the propulsive phase. After the 3-month follow-up, the insole decreased pain intensity and increased foot function. There were significant reductions in the number of participants with hypo-echogenicity of the plantar fascia and that in the apparent US group, which represented a reduction in plantar fascia edema. Positive outcomes from this study supported the use of customized insoles with medial wedges as an effective conservative treatment in patients with PF.

## Data Availability

All data relevant to the study are included in the article or are available as supplementary files. Please ensure that no patient-identifiable data are available.

## Abbreviations

PF: Plantar fasciitis
US: Ultrasonographic
VAS: Visual analog scale
BMI: Body mass index
SLE: Systemic lupus erythematosus
PVC: Polyvinyl chloride
PIG: Plug-In-Gait
OFM: Oxford Foot Model
FFI: Foot function index
MCID: Minimally clinically important differences
GRF: Ground reaction force
SD: Standard deviation
