# Author Correction: Effects of customized insoles with medial wedges on lower extremity kinematics and ultrasonographic findings in plantar fasciitis persons

**DOI:** 10.1038/s41598-024-60480-1

**Published:** 2024-05-03

**Authors:** Suthasinee Thong-On, Pavinee Harutaichun

**Affiliations:** Faculty of Physical Therapy, Mahidol University, 999 Phuttamonthon 4 Road, Salaya, Nakhon Pathom Thailand

Correction to: *Scientific Reports* 10.1038/s41598-023-35862-6, published online 27 May 2023

The original version of this Article contained an error in the Methods section, under the subheading ‘Study design’.

“The research protocols were approved by the center of Ethical Reinforcement for Human Research of Mahidol University (COA No. MU-CIRB 2020/285.2109).”

now reads:

“The research protocols were approved by the center of Ethical Reinforcement for Human Research of Mahidol University (COA No. MU-CIRB 2020/178.0511).”

In addition, under the subheading ‘Ethical approval’,

“The study protocol was approved by the center of Ethical Reinforcement for Human Research of Mahidol University (MU-CIRB 2020/285.2109).”

now reads:

“The study protocol was approved by the center of Ethical Reinforcement for Human Research of Mahidol University (MU-CIRB 2020/178.0511).”

The original Article has been corrected.

